# Reverse‐Engineered Gas‐Fermenting Acetogen Strains Recover Enhanced Phenotypes From Autotrophic Adaptive Laboratory Evolution

**DOI:** 10.1111/1751-7915.70208

**Published:** 2025-08-10

**Authors:** Henri Ingelman, Kurshedaktar Majibullah Shaikh, Kaspar Valgepea

**Affiliations:** ^1^ Institute of Bioengineering, University of Tartu Tartu Estonia

**Keywords:** biochemicals, biofuels, chemostat, gas fermentation, metabolomics, proteomics, reverse genetic engineering

## Abstract

Gas‐fermenting acetogens, such as *Clostridium autoethanogenum*, have emerged as promising biocatalysts capable of converting CO and CO_2_‐containing gases into fuels and chemicals relevant for a circular economy. However, the functionalities of the majority of genes in acetogens remain uncharacterised, hindering the development of acetogen cell factories through targeted genetic engineering. We previously identified gene targets through adaptive laboratory evolution (ALE) that potentially realise enhanced autotrophic phenotypes in *C. autoethanogenum*. In this study, we deleted one of the targets—CLAU_0471 (proposed amino acid permease)—with high mutation occurrence in ALE isolates and extensively characterised the autotrophic growth of strain RE3 in batch bottle and bioreactor continuous cultures. In addition, we characterised two previously reverse‐engineered strains RE1 (deletion of CLAU_3129; putative sporulation transcriptional activator Spo0A) and RE2 (SNP in CLAU_1957; proposed two‐component transcriptional regulator winged helix family). Strikingly, the strains recovered the superior phenotypes of ALE isolates, including faster autotrophic growth, no need for yeast extract, and robustness in bioreactor operation (e.g., low sensitivity to gas ramping, high biomass, and dilution rates). Notably, RE3 exhibited elevated 2,3‐butanediol production, while RE1 performed similarly to the best‐performing previously characterised ALE isolate LAbrini. The three reverse‐engineered strains showed similarities in proteome expression, and bioinformatic analyses suggest that the targeted genes may be involved in overlapping regulatory networks. Our work provides insights into genotype–phenotype relationships for a better understanding of the metabolism of an industrially relevant acetogen.

## Graphical abstract

We used reverse genetic engineering of the gas‐fermenting model‐acetogen *Clostridium autoethanogenum* to recover enhanced phenotypes from autotrophic adaptive laboratory evolution. Reverse‐engineered strains were characterised at systems level and bioinformatics suggest that the targeted genes may be involved in overlapping regulatory networks.

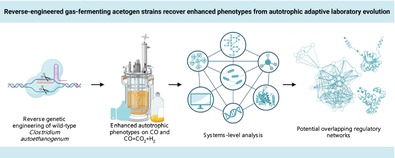

## 1 Introduction

Rapid decarbonisation of energy production is vital for mitigating global warming and maintaining biosustainability on Earth. In addition, the increasingly alarming changes in the global climate have created an urgent need for innovative and sustainable solutions with minimal environmental impact to meet the growing demand for chemicals, fuels, materials, and feed protein. Furthermore, accumulation of waste from the consumption of such products is threatening the stability of ecosystems. One potential route to tackle the dual challenges of sustainable production and recycling of waste is through the application of gas fermentation technologies (Pavan et al. 2022). Gas fermentation uses microbes to convert waste gases (e.g., CO, CO_2_, CH_4_) or syngas (CO + CO_2_ + H_2_) generated from solid waste into valuable fuels (e.g., ethanol), chemicals (e.g., acetone, isopropanol), or feed protein (Marcellin et al. 2022; Pavan et al. 2022). The technology has been commercialised by LanzaTech for the production of ethanol from industrial waste gases using the acetogen *Clostridium autoethanogenum* (Köpke and Simpson 2020).

Acetogens are the preferred biocatalysts for gas fermentation as they can use gas as their sole carbon and energy source (Wood 1991) through the fixation of carbon oxides into acetyl‐CoA via the Wood‐Ljungdahl pathway (WLP) (Ragsdale 2004). Notably, the linear WLP is the most energy‐efficient CO_2_‐fixation pathway known to date (Cotton et al. 2018; Fast and Papoutsakis 2012). Gas fermentation using acetogens is also attractive since variable gas mixtures can be used, the process operates at near‐ambient temperatures and pressures (Bengelsdorf et al. 2018), and is less sensitive to impurities in syngas compared to the chemical Fischer‐Tropsch process (Liew et al. 2016). Acetogens can natively produce, for example, acetate, ethanol, butanediol, lactate, butanol, or hexanol (Drake et al. 2006).

Metabolic engineering has been successfully used in acetogens for the production of over ~50 non‐native chemicals, namely in the industrial cell factory and model‐acetogen *C. autoethanogenum* (Antonicelli et al. 2023; Bae et al. 2022; Daniell et al. 2012; Dykstra et al. 2022; Köpke and Simpson 2020; Liew et al. 2022; Pavan et al. 2022). Yet, targeted improvement of growth, gas uptake, alteration of nutrient requirements, or other native metabolic traits of acetogens is still challenging due to our limited understanding of genotype–phenotype relationships (Pavan et al. 2022). In particular, the large number of genes with unknown or unclear functions poses a challenge for targeted engineering (Brown et al. 2014; Humphreys et al. 2015). One could tackle such challenges in unconventional microbes, such as acetogens, through adaptive laboratory evolution (ALE) experiments, that is, subjection of microbes to growth and selection for driving the evolution of specific traits towards the desired phenotype (Sandberg et al. 2019; Wang 2023). In contrast to rational metabolic engineering, ALE circumvents the need for detailed understanding of the complex metabolic networks of the cell for strain improvement, facilitating optimisation of phenotypes and discovery of new regulatory patterns with less effort (Dragosits and Mattanovich 2013; Mavrommati et al. 2022; Sandberg et al. 2019; Wang 2023; Wu et al. 2022).

In our previous work, we used ALE to obtain seven *C. autoethanogenum* strains that grow faster autotrophically, do not need complex nutrients, and show robustness in operating bioreactor continuous cultures (Ingelman et al. 2024). Briefly, wild‐type *C. autoethanogenum* was subject to three different autotrophic ALE strategies that included serial bottle batch propagation, bioreactor continuous cultures, or a chemical mutagen. ALE isolates exhibited a range of traits and phenotypes, with the strain designated LAbrini displaying a markedly enhanced phenotype. Genetic analysis revealed a multitude of mutations in the isolates, with two genes exhibiting convergent evolution across multiple ALE approaches: CLAU_3129 (putative sporulation transcriptional activator Spo0A) and CLAU_0471 (proposed amino acid permease) (Ingelman et al. 2024).

A minimum of five mutations per strain were detected across three different ALE strategies, and understanding the causality behind these mutations could provide valuable insights into acetogen metabolism. Indeed, the combination of reverse genetic engineering of ALE strains and functional genomics has shown great potential for deciphering genotype–phenotype relationships and providing new genetic engineering targets in both model organisms and unconventional microbes (Kim et al. 2023; Kwon et al. 2022; Long and Antoniewicz 2018; Rychel et al. 2023; Wang 2023; Wu et al. 2022; Xia et al. 2022). For example, Xia and co‐workers were able to design superior *Saccharomyces cerevisiae* producer strains through understanding 2‐phenylethanol growth inhibition (Xia et al. 2022) while Rychel and co‐workers revealed mechanisms behind paraquat tolerance in *Escherichia coli* (Rychel et al. 2023). Such a combined effort of ALE, reverse‐engineering, and functional genomics also improved the understanding of genotype–phenotype relationships behind improved CO tolerance in the gas‐fermenting hydrogenotroph *Cupriavidus necator* (Wickham‐Smith et al. 2023) and in the acetogen *Thermoanaerobacter kivui* (Hocq et al. 2024). A similar approach has led to linking robust CO growth with specific changes in the genome in the gas‐fermenting acetogens *T. kivui* (Jain et al. 2022) and *Eubacterium limosum* (Jin et al. 2022). We thus aimed in this work to link the improved *C. autoethanogenum* phenotypes with specific genes that were mutated during our previous ALE study (Ingelman et al. 2024) to identify causalities and potential epistatic effects behind the detected mutations.

In this study, we reverse‐engineered *C. autoethanogenum* by deleting the gene CLAU_0471 that was mutated in six isolates of our previous ALE work (Ingelman et al. 2024) and extensively characterised the strain in autotrophic bottle and bioreactor continuous cultures together with two other reverse‐engineered strains from the latter ALE study. All three reverse‐engineered strains exhibited significantly improved phenotypes compared to the starting strain of ALE (JA1‐1, wild‐type), demonstrating that these reverse‐engineered strains recover the superior performance of the ALE isolates with faster autotrophic growth in minimal medium and better robustness in continuous cultures. Our work provides insights into genotype–phenotype relationships relevant for a better understanding of the autotrophic metabolism of an industriallyrelevant acetogen.

## 2 Experimental Procedures

### 2.1 Bacterial Strains and Cultivation Conditions

#### 2.1.1 Bacterial Strains

The *C. autoethanogenum* strain JA1‐1 (Abrini et al. 1994) DSM 10061 deposited in the German Collection of Microorganisms and Cell Cultures (DSMZ) was used as the base strain in this study for reverse genetic engineering of strain RE3: JA1‐1 with deletion of CLAU_0471 (amino acid permease). RE3 was constructed using our CRISPR/Cas9‐aided homologous recombination workflow as described before (Nwaokorie et al. 2023). Table S1 lists the primers used and Table S2 lists the strains and plasmids used or constructed in the present study. See Files S1 and S2 for plasmid maps. Other strains that were studied in this work were either genetically engineered strains of JA1‐1 constructed or derivatives of JA1‐1 obtained in our previous work (Ingelman et al. 2024): (1) RE1: JA1‐1 with deletion of CLAU_3129 (sporulation transcriptional activator Spo0A); (2) RE2: JA1‐1 carrying an altered CLAU_1957 (two‐component transcriptional regulator winged helix family) gene (G‐to‐A nucleotide change resulting in glycine to aspartate change in the 94^th^ amino acid); and (3) LAbrini: ALE isolate strain with superior phenotype (Table 1).

**TABLE 1:** Summary of the studied strains.

Strain	Genotype	Source	Gene description
RE1	Deletion of CLAU_3129	Ingelman et al. 2024	Sporulation transcriptional activator Spo0A
RE2	Modified CLAU_1957	Ingelman et al. 2024	Two‐component transcriptional regulator winged helix family
RE3	Deletion of CLAU_0471	This study	Proposed amino acid permease
LAbrini	ALE isolate with 5 mutations	Ingelman et al. 2024	See reference for mutation details

#### 2.1.2 Autotrophic Bottle Cultivations

For autotrophic characterisation of batch growth, RE3 and RE2 were grown in chemically defined PETC‐MES medium (Nwaokorie et al. 2023) with 0.4 g/L of cysteine‐HCl·H_2_O as the reducing agent and either with or without 1.5 g/L of yeast extract (YE). Bottle headspace was pressurised to 140 kPa with either a 60% CO and 40% Ar gas mixture (AS Eesti AGA) or with a syngas mixture (50% CO, 20% CO_2_, 20% H_2_, 10% Ar; AS Eesti AGA). Experiments were carried out with 50 mL medium in 250 mL Schott bottles, incubated horizontally at 37°C with orbital shaking at 120 RPM under strictly anaerobic conditions. Growth was tracked by measuring culture optical density (OD) at 600 nm and cultures were sampled frequently from the exponential phase for maximum specific growth rate (μ_max_) and product yields calculation. μ_max_ was calculated using 3‐to‐6 data points from the exponential phase that yielded a correlation coefficient R^2^ > 0.99 between culture time and ln of OD. Product yields for ethanol, acetate, and 2,3‐butanediol (mmol of product/g of dry cell weight [gDCW]) were calculated during exponential growth by linear regression (R^2^ > 0.9 for RE2, except 0.84 for ethanol for one bioreplicate) or from the difference of two time points (for RE3) between the respective product (mmol/L) and biomass concentrations (gDCW/L) prior to re‐consumption of the products. Data for RE1, RE2 (only + YE medium), LAbrini, and JA1‐1 are from previous studies (Ingelman et al. 2024; Nwaokorie et al. 2023).

#### 2.1.3 Continuous Autotrophic Chemostat Cultivations

Strains RE1 and RE3 were grown on syngas (50% CO, 20% H_2_, 20% CO_2_, and 10% Ar; AS Eesti AGA) in a chemically defined medium (without YE) as described before (Valgepea et al. 2017). Briefly, cells were grown in chemostat continuous cultures under strictly anaerobic conditions at 37°C and at pH 5 (maintained by 5 M NH_4_OH) in 1.4 L Multifors bioreactors (Infors AG) at a working volume of 750 mL connected to a Hiden HPR‐20‐QIC mass spectrometer (Hiden Analytical) for online high‐resolution off‐gas analysis. Antifoam (435530; Sigma‐Aldrich) was continuously added to the bioreactor at 10 μL/h to avoid foaming. Chemostat cultures were operated at variable dilution rates (D) and gas–liquid mass transfer rates (gas flow rate and agitation) to maintain similar steady‐state biomass concentrations (Table 2). Steady‐state results were collected after OD, gas uptake, and production rates had been stable (< 15% variability) for at least three working volumes. Bioreactor off‐gas analysis using a mass spectrometer for determination of specific gas uptake (CO and H_2_) and production rates (CO_2_ and ethanol) (mmol/gDCW/day) and determination of carbon recoveries and balances were carried out as described before (Valgepea et al. 2017). We note that the Faraday Cup detector monitored the intensities of H_2_, CO, ethanol, H_2_S, Ar, and CO_2_ at 2, 14, 31, 34, 40, and 44 amu, respectively, and ethanol stripping and the total soluble CO_2_ fraction in culture broth were also considered for carbon balancing.

**TABLE 2:** Summary of chemostat cultivations with key parameters in this dataset.

Strain	Gas flow rate (mL/min)	Agitation (RPM)	D (day ^−1^)	Biomass concentration (gDCW/L)	Number of replicates
RE1	45	680	0.96 ± 0.01	1.50 ± 0.04	4
RE1	72	810	1.99 ± 0.06	1.58 ± 0.04	4
RE3	25	580	0.53 ± 0.01	1.09 ± 0.15	4
RE3	45	680	1.00 ± 0.04	1.57 ± 0.05	3

*Note:* Gas composition—50% CO, 20% CO_2_, 20% H_2_, 10% Ar. All reported steady‐states are biologically independent. D, dilution rate; gDCW, gram of dry cell weight. Data are average ± standard deviation between bioreplicates.

### 2.2 Genome‐Scale Metabolic Modelling

Genome‐scale metabolic modelling using flux balance analysis (FBA) was performed as described previously (de Lima et al. 2022) with the exception that formate excretion was blocked since it was not detected in exo‐metabolome analysis. We only performed simulations to estimate intracellular metabolic flux rates using maximisation of ATP dissipation as the objective function in FBA.

### 2.3 Analytical Methods

#### 2.3.1 Biomass Concentration and Extracellular Metabolome Analysis

Biomass concentration was determined by measuring culture OD at 600 nm and using the correlation coefficient between culture OD and DCW established at 0.23 using the methodology described before (Peebo et al. 2014). Exo‐metabolome analysis was performed using HPLC as described previously (Ingelman et al. 2024). Briefly, organic acids and alcohols were analysed by a Shimadzu Prominence‐I LC‐2030 plus system using a Rezex ROA‐Organic Acids column, eluted isocratically with H_2_SO_4_, and detected and quantified by a refractive index detector using relevant standards.

#### 2.3.2 Proteome Analysis

Proteome analysis comparing RE3 with JA1‐1 was performed from three biological replicate cultures grown in autotrophic batch bottles on syngas with YE in media (JA1‐1 cannot grow without YE) using exponential‐phase samples (OD ~0.4). Proteome analysis comparing RE1 (four bioreplicates) and RE3 (three bioreplicates) with LAbrini (four bioreplicates) was carried out from steady‐state syngas‐fermenting chemostat cultures (without YE) at D ~1 day^−1^. Sampling, sample preparation, LC–MS/MS analysis using data‐independent acquisition (DIA), and DIA MS data analysis were performed as described before (Nwaokorie et al. 2023). The protein sequence databases of NCBI Genbank CP012395.1 (Humphreys et al. 2015) and NCBI Genbank CP110420 (Ingelman et al. 2024) were used for JA1‐1 and LAbrini comparisons, respectively.

In comparison of RE3 with JA1‐1, we confidently quantitated 30,946 peptides and 2258 proteins across all samples, and 26,483 peptides and 2141 proteins on average within each sample after removing shared peptides from analysis. Only proteins quantified with at least two peptides (2193 across all samples) were used to determine differentially expressed proteins (fold‐change > 2 and *q*‐value < 0.05 after FDR correction (Benjamini and Hochberg 1995)) using the software Perseus (Tyanova et al. 2016) with Student's T‐test. In comparison of RE1 and RE3 with LAbrini, we confidently quantitated 29,564 peptides and 2073 proteins across all samples, and 24,258 peptides and 1994 proteins on average within each sample after removing shared peptides from analysis. Only proteins with at least two peptides (1993 across all samples) were used to determine differentially expressed proteins between RE1 and LAbrini, and RE3 and LAbrini as described above (except with fold‐change > 1.5). Differentially expressed proteins are presented in Tables S5 and S6. Proteomics data have been deposited to the ProteomeXchange Consortium (http://proteomecentral.proteomexchange.org) via the PRIDE partner repository (Perez‐Riverol et al. 2022) with the data set identifier PXD062027. Proteomics data for JA1‐1 and associated files for RE3 vs. JA1‐1 comparison can be found with the data set identifier PXD047330.

## 3 Results and Discussion

We previously used ALE to obtain superior strains of *C. autoethanogenum* and detected 25 mutations across the isolated evolved strains that could have potentially contributed to the enhanced growth phenotypes (Ingelman et al. 2024). In that work, we reverse‐engineered two of the mutations that occurred in multiple isolates in potentially sporulation‐related genes that recovered the improved phenotypes in two strains: RE1 with a *spo0A* deletion (CLAU_3129) and RE2 with a single nucleotide polymorphism (SNP) in CLAU_1957. Additionally, the gene CLAU_0471, annotated as an amino acid permease, appeared as a hotspot for genetic mutations across six isolated strains. This gene displayed an array of mutations, including two SNPs, two deletions, and two premature stop codons. We thus used CRISPR/Cas9‐aided homologous recombination in this work to reverse engineer the CLAU_0471 gene deletion in the starting strain of ALE, *C. autoethanogenum* JA1‐1, to evaluate the effects of mutations in CLAU_0471. Based on the predicted efficiency and minimal off‐targets, two unique guide RNAs (gRNA) were selected to delete the gene, and transformation of either plasmid containing one of the gRNAs into *C. autoethanogenum* using electroporation yielded transformants. While no colonies from gRNA #1 transformation harboured the desired deletion genotype, three of the four screened colonies from gRNA #2 showed only deletion (Figure S1). The CLAU_0471 deletion strain was named RE3.

### 3.1 Performance of Reverse‐Engineered Strains in Autotrophic Batch Cultures

**FIGURE 1:**
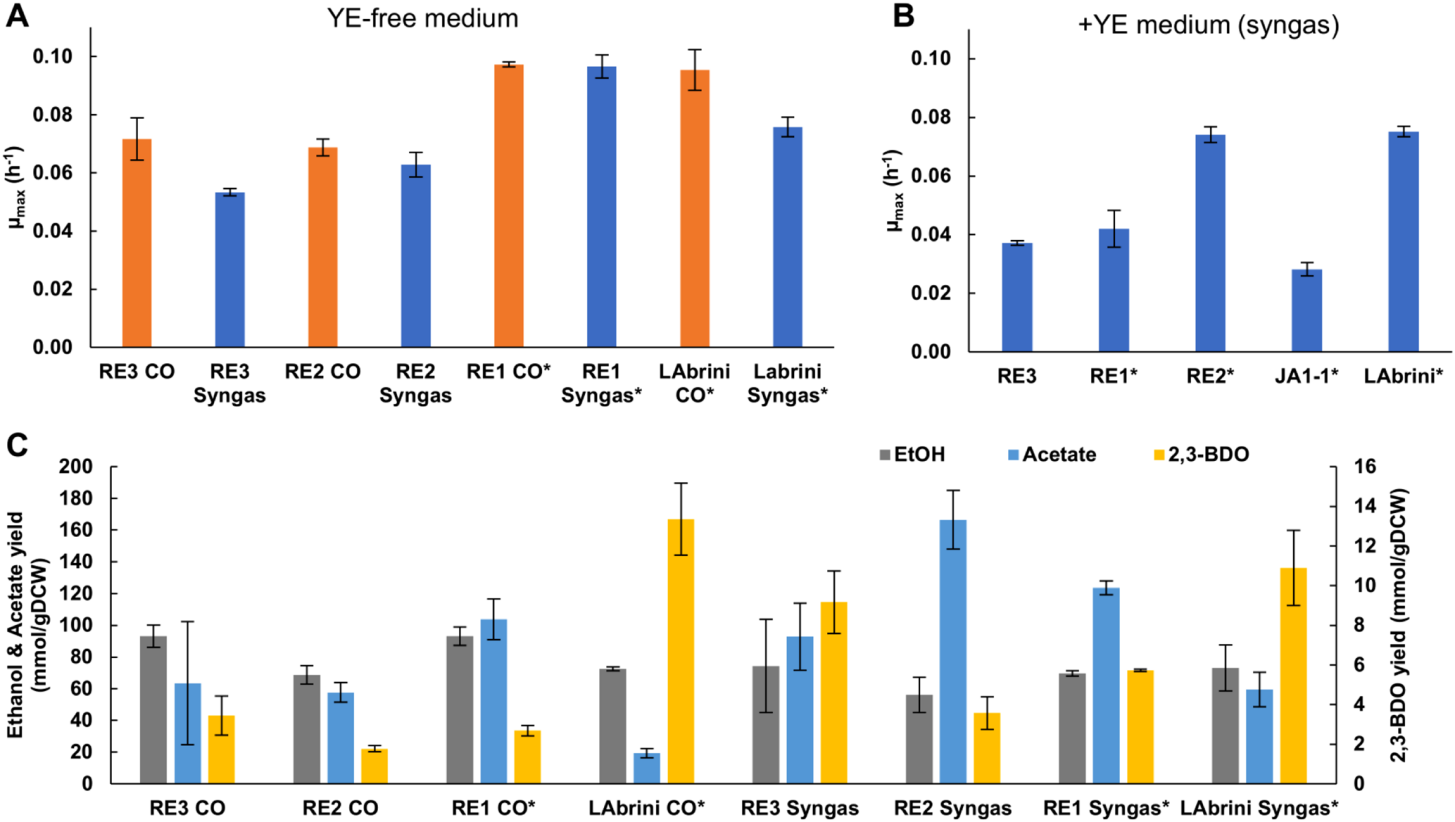
Autotrophic batch bottle characterisation of three reverse‐engineered strains. (A) Comparison of maximum specific growth rate (μ_max_) of studied strains on YE‐free medium. Orange bar denotes CO feed gas, blue syngas. The wild‐type strain JA1‐1 does not grow in YE‐free medium, therefore it was not included in this comparison. Bars show average ± standard deviation between three bioreplicates, except two bioreplicates for LAbrini syngas and RE1 syngas (YE‐free medium). (B) μ_max_ of studied strains on medium with YE on syngas. Bars show average ± standard deviation between three bioreplicates. (C) Production yields (mmol/gDCW) of growth by‐products of studied strains on YE‐free medium. Bars show average ± standard deviation between three bioreplicates, except two bioreplicates for LAbrini CO and RE1 syngas. Asterisk behind strain name denotes previously published data (Ingelman et al. 2024; Nwaokorie et al. 2023). gDCW, gram of dry cell weight; 2,3‐BDO, 2,3‐butanediol.

We compared strain RE3 in autotrophic batch bottle cultures with the starting strain of ALE (JA1‐1), the superior strain LAbrini obtained through ALE, and the previously reverse‐engineered strains RE1 and RE2 (Ingelman et al. 2024). We grew RE3 and RE2 in this work while the rest of the data are from our previous works (Ingelman et al. 2024; Nwaokorie et al. 2023; denoted by asterisk on figures). Experiments conducted without yeast extract (YE) demonstrated that RE3 can grow without YE and with μ_max_ of 0.072 ± 0.007 and 0.053 ± 0.001 on CO and syngas, respectively, which is similar to RE2. However, RE3 grew 1.3‐fold and 1.8‐fold slower than RE1 and 1.3‐fold and 1.4‐fold slower than LAbrini on CO and syngas, respectively (Figure 1A). In addition, RE3 had a 1.4‐fold higher μ_max_ on CO than on syngas, similar to LAbrini (1.3‐fold higher), while this was not observed for RE1 and RE2 as they displayed μ_max_ of ~0.1 and ~0.06 on both gases, respectively (Ingelman et al. 2024). We note that the wild type strain JA1‐1 does not grow in YE‐free medium. When comparing the reverse‐engineered strains to the starting strain of ALE (JA1‐1) with YE‐containing medium on syngas (Figure 1B), only RE2 grew significantly faster than JA1‐1 (μ_max_ of 0.074 ± 0.003 vs. 0.028 ± 0.002 for JA1‐1). Strikingly, RE1 and RE3 grew 2.3‐fold and 1.4‐fold slower with YE than without it (Figure 1). Therefore, it seems that the presence of YE in the growth medium seems to have a trade‐off on μ_max_ for RE1 and RE3. Nevertheless, the reverse‐engineered strains were able to grow faster than JA1‐1 (with YE) and without YE, demonstrating that the targeted single mutations could recover the faster growth phenotype of ALE isolates (Ingelman et al. 2024).

Analysis of growth by‐products for the YE‐free media bottle batch experiments (Figure 1C) showed significantly higher production of 2,3‐butanediol (2,3‐BDO) for all three reverse‐engineered strains (2‐to‐2.7‐fold) when grown on syngas compared to CO (*p* values = 0.001–0.02), while no significant difference was seen for LAbrini (Ingelman et al. 2024; Nwaokorie et al. 2023). While ethanol production was 1.3‐fold higher on CO for RE1 (*p* value = 0.01) and acetate production was 2.9‐fold higher on syngas for RE2 (*p* value = 0.001), acetate/ethanol ratios (yield/yield) were higher by 1.6‐ and 3.7‐fold for RE1 and RE2, respectively on syngas (*p* value of 0.02 for RE1 and 0.01 for RE2) (Figure 1C). This is surprising as the H_2_ in syngas is expected to support increased synthesis of reduced products compared to growth on CO only (see 3.2. for discussion). When comparing reverse‐engineered strains, RE2 exhibited overall a lower yield of 2,3‐BDO compared to RE3 and RE1 on both gases with 1.5‐ and 1.6‐fold lower yield on CO and syngas compared to RE3, respectively (no difference between RE3 and RE1). In terms of ethanol yields, production was similar between RE3 and RE1 (*p* values of 0.99 and 0.85) while both RE3 and RE1 produced ~1.4‐fold more ethanol on CO than RE2 (*p* values = 0.01). Notably, RE2 produced 1.8‐ and 1.4‐fold more acetate on syngas compared to RE3 and RE1, respectively (*p* values of 0.01 and 0.05, respectively). When comparing yields to LAbrini, both RE1 and RE3 showed 1.3‐fold higher ethanol yields on CO (*p* values of 0.01 and 0.03) while the 2,3‐BDO yields remained lower for all three engineered strains on CO (*p* values = 0.001–0.004). Additionally, RE1 and RE2 produced more acetate than LAbrini on both gases (*p* values = 0.002–0.005) that also translated into 2–4‐fold higher acetate/ethanol ratios (*p* values = 0.01–0.02) (Figure 1C). Similar to the ALE isolates (Ingelman et al. 2024), all reverse‐engineered strains displayed heterogeneity in by‐product formation. However, single mutations alone did not reproduce the by‐product yields of ALE isolates, highlighting that ALE isolate phenotypes emerge from the combined effect of all accumulated mutations (Ingelman et al. 2024).

All three studied reverse‐engineered strains were able to grow in autotrophic batch cultures without YE, which is required for wild‐type strain JA1‐1 (see last paragraph of 3.4 for potential reasoning). Previously, *C. ljungdahlii and C. autoethanogenum* have been adapted to grow without YE (Martin et al. 2015; Schulz et al. 2023) while our results show that YE‐free growth could be achieved with single‐gene targeted modifications. Additionally, all the reverse‐engineered strains grew significantly faster without YE compared to the wild‐type strain with YE, which further supports the benefits of reverse‐engineering mutations uncovered in ALE studies (Dragosits and Mattanovich 2013; Ingelman et al. 2024).

### 3.2 Reverse‐Engineered Strains Exhibit Enhanced Solvent Production and Performance in Autotrophic Continuous Bioreactor Cultures

In addition to the comparison of strains in bottle batch cultures, we next compared RE1, RE3, and LAbrini in syngas‐fermenting chemostat cultures to obtain steady‐state data on minimal medium. Strain JA1‐1 was excluded from this comparison as it cannot grow in the absence of YE. In this work, we grew RE3 cultures at dilution rates (D) 0.5 and 1 day^−1^ and RE1 at D = 1 and 2 day^−1^ while LAbrini data at D = 1 and 2 day^−1^ are from previous works (Ingelman et al. 2024; Nwaokorie et al. 2023; denoted by asterisk on figures). As RE3 turned out to be less robust in terms of bioreactor operation compared to RE1 and LAbrini, operating a culture at D = 2 day^−1^ was not attempted. Cultures of different strains at the same D were operated at similar gas–liquid mass‐transfer conditions (i.e., biomass concentrations) to have a fair comparison between strains (see Section 2. Experimental Procedures).

**FIGURE 2:**
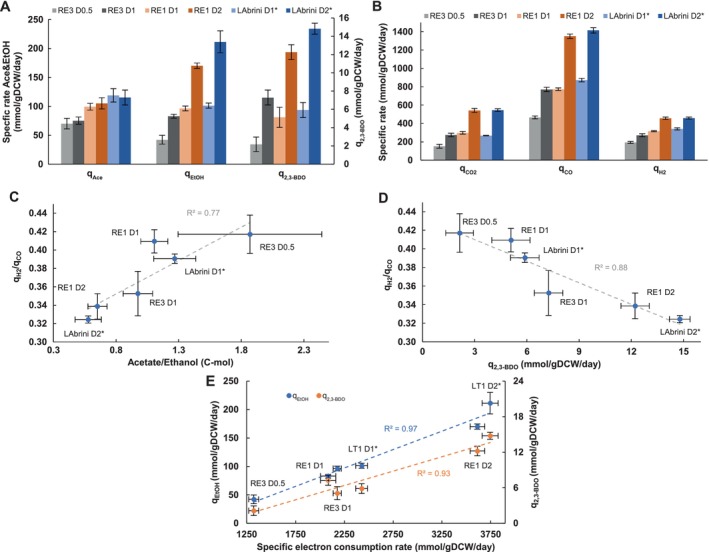
Performance of RE3, RE1, and LAbrini in syngas‐fermenting chemostat cultures. (A) Specific by‐product production rates. (B) Specific gas uptake and production rates. (C) Correlation between q_H2_/q_CO_ and acetate/ethanol (C‐mol) ratios. (D) Correlation between q_H2_/q_CO_ ratio and specific 2,3‐BDO production rate. (E) Correlation between q_EtOH_ and q_2,3‐BDO_, and specific electron consumption rate ((q_H2_ and q_CO_ summed) × 2). RE3 D = 1 day^−1^ is denoted with an open symbol. The number following D (dilution rate) denotes the D value in day^−1^. Bars show average ± standard deviation between bioreplicates (see Section 2. Experimental Procedures for details). Asterisk behind the name denotes previously published data (Ingelman et al. 2024). EtOH, ethanol; 2,3‐BDO, 2,3‐butanediol; Ace, acetate; q, specific production rate, except specific uptake for CO and H_2_.

When examining by‐product formation, there was no distinct difference between acetate and ethanol at D = 1 day^−1^ (Figure 2A) for the studied strains. Notably, RE3 showed a 1.4‐fold elevated specific 2,3‐BDO production rate (q_2,3‐BDO_; *p* value = 0.036). At D = 2 day^−1^, strain RE1 exhibited 19% lower q_EtOH_ (170.24 ± 4.57 vs. 211.4 ± 18.9) and 18% lower q_2,3‐BDO_ (12.22 ± 0.81 vs. 14.79 ± 0.6) in comparison to LAbrini (p‐values of 0.006 and 0.002 for q_EtOH_ and q_2,3‐BDO_, respectively). This is consistent with differences in metabolite concentrations in RE1 and LAbrini (Figure S2). Interestingly, the increase in q_Ace_ was not proportional to the increase of D for both reverse‐engineered strains (RE1 and RE3).

In terms of gas consumption and production, the performance of RE1 was very similar to LAbrini at both D = 1 and 2 day^−1^ (Figure 2B). As expected for CO‐limited chemostats, the specific CO consumption rate (q_CO_) increased by 1.7‐fold, which is nearly proportional to the increase in D (Figure 2B). However, the 1.4‐fold increase of the specific consumption rate of H_2_ (q_H2_) was not proportional to D as seen before (de Lima et al. 2022). At the same time, the trend between the total specific electron consumption rate (sum of H_2_ and CO) and the acetate/ethanol ratio (Figure S3) is consistent with the latter study: cultures growing at higher D are directing less carbon towards acetate. For RE3, genome‐scale metabolic flux simulations using FBA showed that all the H_2_ was used up for reducing CO_2_ to format in the WLP (Figure S4; Tables S3 and S4). Notably, we observed a positive correlation (*R* ^2^ = 0.77) between acetate/ethanol (C‐mol) and q_H2_/q_CO_ ratios (Figure 2C) and a strong negative correlation between q_2,3‐BDO_ and q_H2_/q_CO_ (Figure 2D) (*R* ^2^ = 0.88). This indicates that less reduced products (i.e., ethanol and 2,3‐BDO) are produced with a higher supply of carbon‐free redox (i.e., H_2_), at least when comparing syngas with CO. Intriguingly, this is not consistent with previously published results for *C. autoethanogenum* grown on syngas (50% CO, 20% CO_2_ 20% H_2_) and CO + H_2_ (15% CO, 45% H_2_) (Valgepea et al. 2018) where the authors demonstrated that providing more carbon‐free redox (as H_2_) directs more carbon into reduced products (including ethanol). Regarding specific electron consumption rates in this dataset, there is a strong correlation with q_EtOH_ (*R* ^2^ = 0.97) and q_2,3‐BDO_ (*R* ^2^ = 0.93) (Figure 2E), which is in agreement with previous analysis (Allaart et al. 2023). This further highlights the higher 2,3‐BDO production in RE3 as its D = 1 day^−1^ data‐point clearly deviates from the general trend of increasing q_2,3‐BDO_ with increasing D (Figure 2D). Interestingly, flux simulations showed a lower maintenance energy and a lower percentage of the latter from total ATP production in RE3 (Figure S4; Tables S3 and S4).

**FIGURE 3:**
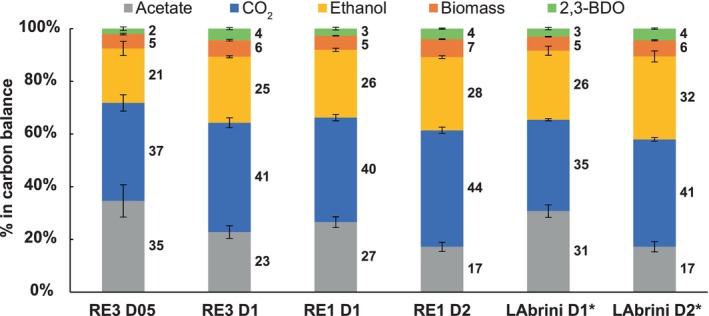
Carbon balances of RE3, RE1, and LAbrini syngas‐fermenting chemostat cultures. The number following D (dilution rate) denotes D value in day^−1^. Carbon recoveries were normalised to 100% to have a fair comparison of carbon distributions between strains and conditions. Bars show average ± standard deviation between bioreplicates (see Section 2. Experimental Procedures for details). Asterisk behind name denotes previously published data (Ingelman et al. 2024). 2,3‐BDO, 2,3‐butanediol.

Carbon balance analysis shows that RE1 closely replicates LAbrini while showing increased carbon flow into CO_2_ and slightly lower ethanol at D = 2 day^−1^ (Figure 3). When comparing RE3 to RE1 and LAbrini at D = 1 day^−1^, RE3 displays lower acetate compared to both strains and a higher percentage of carbon towards CO_2_ compared to LAbrini. The effect of increasing D on the carbon balance is similar for the three strains: for example, increasing D diverted more carbon into solvents and CO_2_, which is in line with the fact that the acetate/ethanol ratio decreases at higher D (Figure S2).

The performance of RE3 and RE1 in batch and continuous cultures indicates that genes under higher selection pressure in ALE are potentially attractive targets for targeted genetic engineering (Dragosits and Mattanovich 2013). Importantly, the reverse‐engineered strains are notably more robust compared to most isolates from ALE that harboured multiple mutations (Ingelman et al. 2024). For example, we previously faced recurring difficulties in achieving stability of the bioreactor continuous cultures for most ALE isolates (Ingelman et al. 2024) while achieving steady state for RE1 even at D = 2 day^−1^ was straightforward. These observations might suggest that we avoided the epistatic effects often present in ALE‐derived strains (Carpenter et al. 2023; Chou et al. 2011; Khan et al. 2011) by reverse‐engineering specific mutations detected in ALE into strains RE1, RE2, and RE3.

### 3.3 Similarities in Proteome Responses of Reverse‐Engineered Strains RE1 and RE3

We next characterised proteome expression patterns for the three chemostat‐grown strains by comparing the reverse‐engineered strains RE1 and RE3 to the superior ALE‐derived strain LAbrini at D = 1 day^−1^. We identified 107 differentially expressed proteins (DEP) (fold‐change > 1.5 and *q* < 0.05) between RE1 and LAbrini, 46 between RE3 and LAbrini, and 24 shared DEPs between RE1 and RE3 (Table S5). The number of DEPs (with fold‐change > 2) was considerably higher for the comparison of RE1 and RE2 with JA1‐1 batch cultures in our previous work (Ingelman et al. 2024), indicating higher similarity between the reverse‐engineered strains and LAbrini. Indeed, the proteome of JA1‐1 batch cultures differs clearly from the proteomes of LAbrini and reverse‐engineered strains (Figure S5). Compared to LAbrini, CLAU_0471 (LABRINI_02360; i.e., gene deleted in RE3) and carbamate kinase (10405) were not detected in RE3 while a transcriptional regulator (05890) was not detected in RE1. Conversely, we detected the expression of a transcriptional regulator (00325) and alcohol dehydrogenase (Adh) (05230) in RE3 but not in LAbrini.

In contrast to the many sporulation‐related proteins found to be differentially expressed in RE1 compared to JA1‐1 (ALE starting strain) batch cultures (Ingelman et al. 2024), the expression of sporulation‐related proteins was indifferent between RE1 and LAbrini in chemostats (Table S5). Interestingly, the histidine kinase (16380; CLAU_3194) potentially involved in sporulation networks (Ingelman et al. 2024) was up‐regulated two‐fold in RE3 compared to LAbrini (Table S5). This kinase was mutated in all isolates of one of the three ALE strategies previously employed (Ingelman et al. 2024) and detected as a DEP in both RE1 and RE2 compared to JA1‐1 batch cultures (Ingelman et al. 2024). Comparison of proteome expression of RE3 and JA1‐1 autotrophic batch cultures also detected LABRINI_16380 as a DEP (Table S6). It is also noteworthy that numerous proposed histidine kinases were detected as DEPs in all batch proteomics comparisons of reverse‐engineered strains with JA1‐1 (fold‐changes 2.3‐to‐3.5 in RE3 vs. JA1‐1; Table S6; Ingelman et al. 2024).

For both RE1 and RE3 chemostat data, we noted 4‐fold upregulation of the alcohol dehydrogenase (Adh4; 09125) with highest protein expression in *C. autoethanogenum* (Valgepea et al. 2022) and a 3‐fold upregulation for another Adh (18005) in RE3 (Table 1). Within the carbon‐fixing WLP pathway, ~2‐fold upregulation of formate‐tetrahydrofolate ligase (Fhs; 08015) and cyclodeaminase/cyclohydrolase family protein (08010) in RE3, and of a formate dehydrogenase (13975) in RE1 was detected (Table 3, Table S5). At the same time, a formate dehydrogenase (00410), its preceding 4Fe‐4S cluster protein (00405), and a carbon monoxide dehydrogenase (08025) were downregulated 1.6‐to‐1.8‐fold in RE3. Additionally, various oxidoreductases were differentially expressed in both strains (e.g., 04430, 00385, 05235; Table S5) with only one showing a similar trend: 6‐ or 7.5‐fold downregulation of a proposed FAD‐dependent oxidoreductase (15005) for RE1 or RE3, respectively. Lastly, in RE1 we detected strong upregulation of gluconeogenesis enzymes (16255, 16260; 10‐fold), a sigma54‐dependent transcriptional regulator (05770, 4‐fold); and downregulation of the aldehyde ferredoxin oxidoreductase AOR1 (00445; 2‐fold) and the carbon starvation protein A (07885; 3‐fold). The latter may indicate either changes in sensing carbon limitation or in sporulation processes (Kevorkian et al. 2016) in RE1 though we could not detect sporulation in our previous work (Ingelman et al. 2024). Similarly, a *T. kivui* isolate evolved to tolerate and grow robustly on pure CO exhibited a notable upregulation of numerous sporulation‐related genes (Hocq et al. 2024). The authors propose that despite the inability of *T. kivui* to form mature spores, the isolate could employ part of the sporulation signalling cascade to cope with CO toxicity.

**TABLE 3:** Notable differentially expressed proteins (DEPs) in RE1 and RE3 strains compared to LAbrini in syngas‐fermenting chemostat cultures.

Protein ID	Description of protein product	RE1 vs. LAbrini			RE3 vs. LAbrini		
		*q*‐value	Log_2_ FC	FC	*q*‐value	Log_2_ FC	FC
*Ethanol production*							
LABRINI_18005	Iron‐containing alcohol dehydrogenase				0.02	1.6	2.9
LABRINI_09125	Iron‐containing alcohol dehydrogenase	0.01	1.9	3.6	0.02	2.0	4.0
LABRINI_00445	Aldehyde ferredoxin oxidoreductase	0.02	−1.1	2.1			
*C*_*1*_ *‐fixing WLP pathway*							
LABRINI_00405	4Fe‐4S dicluster domain‐containing protein	0.02	−0.7	1.6	0.04	−0.8	1.7
LABRINI_00410	Formate dehydrogenase subunit alpha				0.03	−0.8	1.8
LABRINI_08010	Cyclodeaminase/cyclohydrolase				0.01	0.9	1.8
LABRINI_08015	Formate—tetrahydrofolate ligase				0.03	0.9	1.9
LABRINI_08025	Carbon‐monoxide dehydrogenase catalytic subunit				0.04	−0.7	1.6
LABRINI_13975	Formate dehydrogenase subunit alpha	0.01	1.1	2.1	0.05	1.1	2.2

*Note:* Positive FC means up‐regulation, negative down‐regulation. See Table S5 for all differentially expressed proteins (DEPs).

Abbreviations: FC, fold‐change; ID, identifier.

### 3.4 Reverse‐Engineered Genes May Be Involved in Overlapping Regulatory Networks

Strain RE3 carrying CLAU_0471 deletion showed significant phenotypes, such as lower μ_max_ during growth with YE compared to its absence, and high 2,3‐BDO production in both bottle batch and continuous culture experiments. It is unclear why an amino acid permease (as CLAU_0471 is annotated based on gene sequence homology) would obtain multiple mutations, including large deletions, across two different ALE strategies during growth on minimal medium (Ingelman et al. 2024). However, alternative functions for CLAU_0471 emerge from bioinformatic searches using its protein sequence. For instance, DeepFRI (Gligorijević et al. 2021) prediction based on protein structures expands CLAU_0471 function to a transmembrane (ion) transporter. Regardless of the actual function of the predicted membrane protein, its abundance is very low: ~20–35‐fold lower than for carbon monoxide dehydrogenase (CODH) (Valgepea et al. 2022). Thus it is unlikely that evolutionary pressures in ALE (Ingelman et al. 2024) selected against this protein to save energy or membrane surface area from lack of its synthesis. According to CDART (Geer et al. 2002), the PotE domain found in CLAU_0471 is also present in proteins with proposed histidine kinase and signal transduction histidine kinase functions in various other taxa, including some *Clostridium* species. This is notable as histidine kinases can regulate sporulation and metabolism in Clostridia (Xin et al. 2020; Xu et al. 2015) and RE1 and RE2 were engineered with mutations in potentially sporulation‐related genes that restored the superior phenotypes acquired in ALE (Ingelman et al. 2024).

Transcription start site data of *C. autoethanogenum* (de Souza Pinto Lemgruber et al. 2019) indicates that CLAU_0471 does not belong to an operon. Based on the STRING protein–protein interactions database (Szklarczyk et al. 2015), genes up‐ and down‐stream of CLAU_0471 appear to have various functions related either to metabolism or cell signalling. For instance, two translated proteins downstream on the same strand are related to energy and pyrimidine metabolism: CLAU_0470, a SPFH domain‐containing protein within “ATP synthesis” and “Oxidative phosphorylation” local clusters; and CLAU_0469, a proposed sugar kinase within the “Pyrimidine metabolism” local cluster. The gene upstream on the same strand (CLAU_0473), which encodes a proposed methyl‐accepting chemotaxis protein, is associated with biological processes related to “Signal transduction” and “Chemotaxis” according to STRING.

**FIGURE 4:**
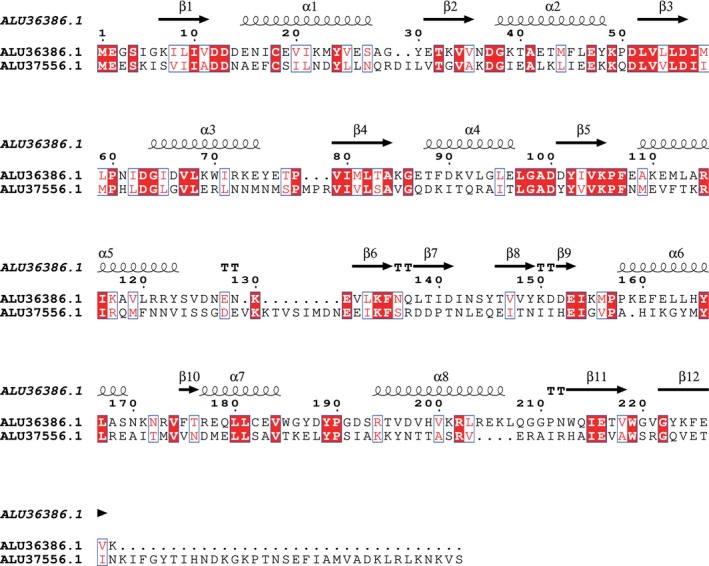
Secondary structure alignment of CLAU_1957 and Spo0A protein sequences. Representation was done with ESPript 3.0, see ESPript webpage for details (https://espript.ibcp.fr). Perfectly conserved residues are highlighted with white font plus red box and well‐conserved residues are denoted with red font plus blue box. β‐sheets, α‐helices, and β‐turns are highlighted as arrows, springs and bold TT, respectively. ALU36386.1, CLAU_1957 protein sequence; ALU37556.1; Spo0A protein sequence.

The two other reverse‐engineered strains RE2 and RE1 in this study have genetic modifications in CLAU_1957 and Spo0A, respectively. According to STRING, both genes are predicted to have more than 10 functional partners: Spo0A is related to sporulation while CLAU_1957 is part of “Two‐component regulatory system”, and “Phosphorelay sensor kinase activity” local network clusters. DeepFRI and ProteinInfer predictions for CLAU_1957 are consistent with STRING as it is predicted to be involved in phosphorelay activities (Ingelman et al. 2024). Although it is recognised that Spo0A serves as a master regulator of sporulation in Clostridium species (Dürre 2016; Kolek et al. 2017), we were unable to detect sporulation in wild‐type *C. autoethanogenum* or the effects of Spo0A deletion on the latter phenotype in our previous work (Ingelman et al. 2024). Interestingly, protein sequences of CLAU_1957 and Spo0A share similarity (27.6% identity with 97% query coverage; E value 2e^−18^) (Altschul et al. 1997) and significant amount of conserved residues in their secondary structures (Figure 4). It is noteworthy that CLAU_1957 gene sequence also shares some partial similarity with a proposed histidine kinase (CLAU_3194; which acquired mutations in our previous study (Ingelman et al. 2024)). Protein CLAU_3194 is also predicted to belong to a phosphorelay signal transduction two‐component system according to STRING.

**FIGURE 5:**
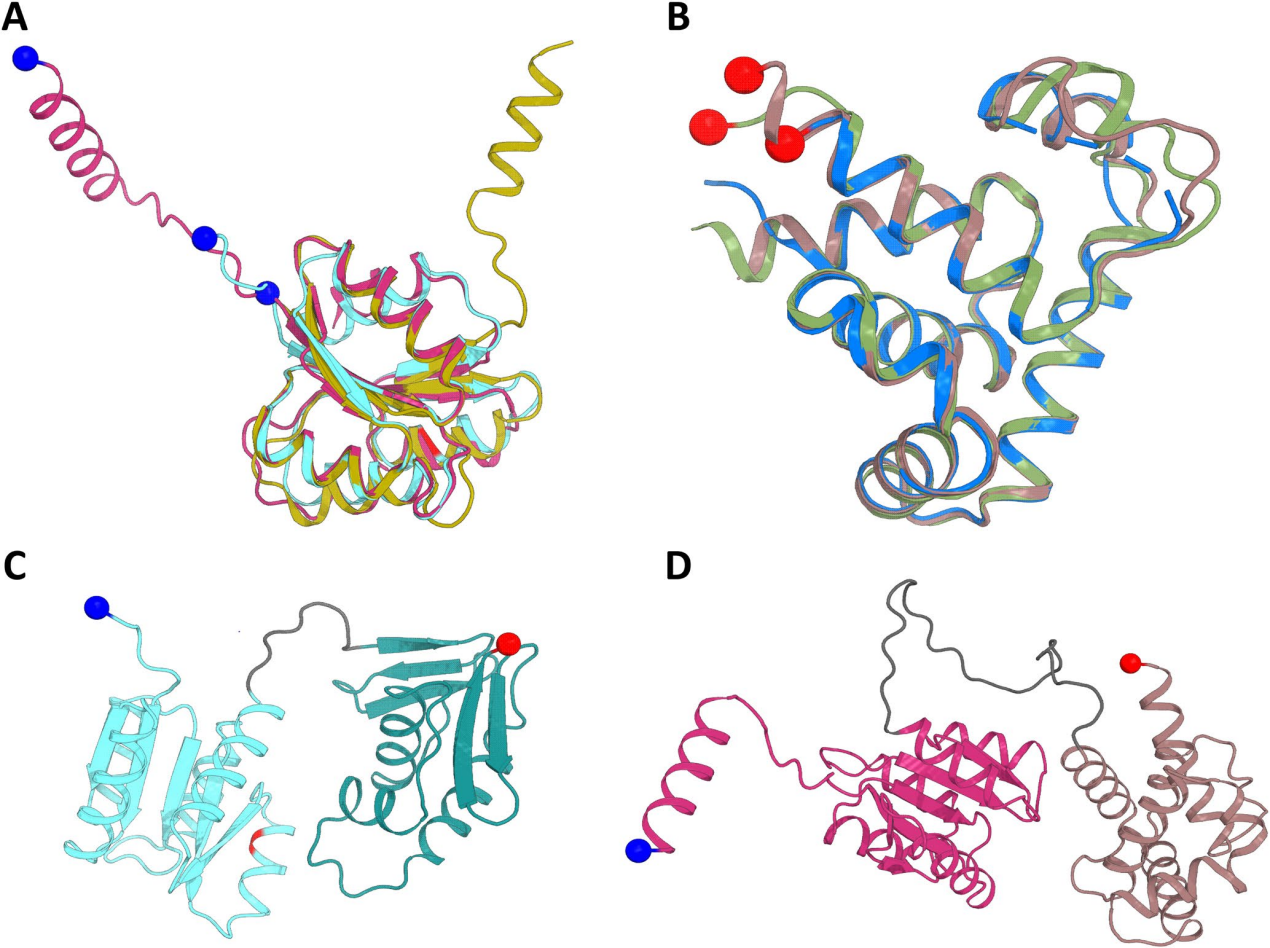
Comparison of Alphafold 2 predicted domains of CLAU_1957 and Spo0A with their closest structural homologues. (A) Superposition of the N‐terminal domains of C. *autoethanogenum* Spo0A Alphafold 2 model (Uniprot no. U5RXI2; shown residues 1–144; pink), CLAU_1957 Alphafold 2 model (Uniprot no. U5RXC5; shown residues 1–123; blue) and *Geobacillus stearothermophilus* Spo0A structure determined by X‐ray diffraction (PDB no 1DZ3, (Lewis et al. 2000); shown residues 1–124; yellow). Superposition of U5RXC5 to U5RXI2 resulted in an RMSD of 0.84 Å for 92 Cα aligned; superposition of 1DZ3 to U5RXI2 resulted in an RMSD of 0.84 Å for 86 Cα aligned. (B) Superposition of C‐terminal domains of C. *autoethanogenum* Spo0A Alphafold 2 model (Uniprot no. U5RXI2; shown residues 174–291; brown) with *B. subtilis* Spo0A (PDB no. 1LQ1, (Zhao et al. 2002); shown residues 150–264; blue) and *Geobacillus stearothermophilus* Spo0A (PDB no. 1FC3, Lewis et al., 2002; shown residues 140–258; green) structures determined by X‐ray diffraction. Superposition of 1LQ1 or 1FC3 on U5RXI2 resulted respectively in an RMSD of 0.38 Å for 92 Cα aligned or an RMSD of 0.38 Å for 94 Cα aligned. (C) AlphaFold 2 proposed model of C. *autoethanogenum* CLAU_1957 (Uniprot no. U5RXC5) with mutation in RE2 strain in phosphorelay signal receiver domain highlighted in red (in 94^th^ amino acid, glycine exchanged for an asparagine). The N‐terminal half is shown in cyan, the linker in grey and the C‐terminal half in dark teal. (D) AlphaFold 2 proposed model of C. *autoethanogenum* Spo0A (Uniprot no. U5RXI2) with the N‐terminal half coloured in pink, the linker shown in grey and the C‐terminal half in brown. The N and C termini are highlighted as blue and red spheres, respectively. Sequence alignment was performed using PyMOL version 2.2.0 (Schrödinger LLC).

When comparing CLAU_1957 and Spo0A sequences against a reported list of conserved sporulation genes in all spore‐forming bacilli and Clostridia (Galperin et al. 2012), both proteins share significant similarity (CLAU_1957—31.7% identity with 49% query coverage; E value 3e^−15^; Spo0A—57.6% identity with 98% query coverage; E value e^−108^) with the *B. subtilis* Spo0A protein (NCBI ref. no. NP_390302.1). Intriguingly, comparison of the predicted structures of CLAU_1957 and Spo0A (Jumper et al. 2021) with the only fully experimentally determined structure of Spo0A ( *Geobacillus stearothermophilus* , Lewis et al. 2000) revealed that the N‐terminal subdomain of all three proteins is highly conserved (Figure 5A). It is notable that the introduced mutation in RE2 (Gly to Asp change in 94^th^ position) is in the same subdomain, the phosphorelay signal receiver domain (Diallo et al. 2021). Additionally, we uncovered that the C‐terminal subdomain (the DNA binding domain; Brown et al. 1994) of the proposed *C. autoethanogenum* Spo0A structure shares high similarity with experimentally determined structures from *B. subtilis* and *Geobacillus stearothermophilus* (Figure 5B). Furthermore, the N‐terminal subdomain of CLAU_1957 is conserved when compared to the closest analogue with a determined structure ( *Mycobacterium tuberculosis* “Sensory transduction protein regX3”; data not shown). This suggests that both CLAU_1957 and Spo0A may have been relatively well conserved components of sporulation systems in a common ancestor. In conclusion, our results indicate that protein products of several mutated genes in our previous ALE experiments (Ingelman et al. 2024) and examined in this study may be part of overlapping regulatory networks or have carried signalling protein functions in common evolutionary ancestors. In line with this, the blastP‐based multiple sequence alignment revealed that the three mutated genes in RE1, RE2, and RE3 exhibit moderate conservation across the Clostridia class (Figure S6). Both highly conserved regions and variable hotspots were identified, suggesting possible partial functional divergence among orthologs. Although, to the best of our knowledge, no direct links between these genes and biosynthesis capacities of biomass monomers in acetogens have been described, the potential regulatory network(s) involving the genes studied in this work could facilitate activation of pathways or reactions realising biosynthesis of all biomass monomers instead of the need for their external supplementation, for example, by YE. In order to further test these hypotheses, additional studies employing a combination of multiple gene edits, including disruption and partial deletions, are required.

## 4 Conclusions

Our work is significant as we have validated the effects of mutations in three genes observed in our previous ALE study (Ingelman et al. 2024) by constructing RE3 and characterising all three reverse‐engineered strains RE1, RE2, and RE3. The various single gene edits we introduced were able to reproduce the superior phenotypes of ALE isolates in both bottle batch and bioreactor continuous cultures, such as faster, more robust, and YE‐free growth under autotrophic conditions. Importantly, these results suggest that convergent evolution across different ALE strategies resulted in similar phenotypes but through different genetic pathways, potentially with overlapping regulatory networks. Further studies are needed to explore potential cumulative or epistatic effects from combining mutations of the tested gene targets.

## Author Contributions

**Henri Ingelman:** conceptualization, methodology, formal analysis, investigation, writing – original draft, writing – review and editing. **Kurshedaktar Majibullah Shaikh:** conceptualization, methodology, investigation, writing – review and editing. **Kaspar Valgepea:** conceptualization, methodology, formal analysis, investigation, resources, writing – original draft, writing – review and editing, funding acquisition, project administration, supervision.

## Conflicts of Interest

The authors declare no conflicts of interest.

## Supporting information


**Figure S1:** PCR screening of transformant colonies for CLAU_0471 deletion.

**Figure S2:** Biomass and by‐product concentrations of RE3, RE1, and LT1 autotrophic chemostat syngas cultures.

**Figure S3:** Correlation between acetate/ethanol (C‐mol) ratio and total specific electron consumption rate per biomass ((qH2 and qCO summed) × 2) across the whole bioreactor dataset.

**Figure S4:** In silico estimation of intracellular metabolic fluxes of central metabolism in syngas‐fermenting RE1, RE3, and LAbrini chemostats (see Figures 2 and 3 for experimental data).

**Figure S5:** Principal component analysis (PCA) of protein MS intensities for the reverse‐engineered strains RE1, RE2, RE3, and LAbrini and JA1–1.

**Figure S6:** Multiple sequence alignment of proteins reverse‐engineered in this study with homologous proteins among Clostridia species.

**Table S1:** Primers used in this study.

**Table S2:** Strains and plasmids used for genetic engineering in this study.

**Table S3:** Summary of in silico estimation of intracellular metabolic fluxes with the GEM iCLAU786.

**Table S4:** Complete results of in silico estimation of intracellular metabolic fluxes with the GEM iCLAU786.

**Table S5:** Differentially expressed proteins between RE1, RE3 and LAbrini in syngas‐fermenting chemostat cultures.

**Table S6:** Differentially expressed proteins between RE3 and JA1‐1 in syngas bottle batch cultures.

**File S1:** mbt270208‐sup‐0013‐Supplementary File 1.dna.

**File S2:** mbt270208‐sup‐0014‐Supplementary File 2.dna.

## Data Availability

Proteomics data have been deposited to the ProteomeXchange Consortium (http://proteomecentral.proteomexchange.org) via the PRIDE partner repository (Perez‐Riverol et al. 2022) with the data set identifier PXD062027.
