# Supplementary material for: Reverse‐Engineered Gas‐Fermenting Acetogen Strains Recover Enhanced Phenotypes From Autotrophic Adaptive Laboratory Evolution
Source: Microb Biotechnol. 2025 Aug 10;18(8):e70208. doi: 10.1111/1751-7915.70208 (PMC12335938; doi:10.1111/1751-7915.70208)
Supplement: Supplementary file 1 — Figure S1: PCR screening of transformant colonies for CLAU_0471 deletion. [file MBT2-18-e70208-s012.pdf]

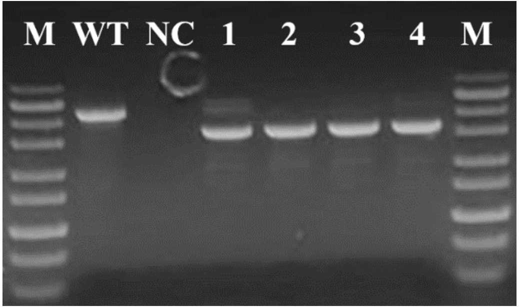

Figure S1. PCR screening of transformant colonies for CLAU_0471 deletion. M, DNA ladder; WT, starting wild-type strain JA1-1; NC, negative control; 1–4, four screened transformant colonies.
