# Supplementary material for: Reverse‐Engineered Gas‐Fermenting Acetogen Strains Recover Enhanced Phenotypes From Autotrophic Adaptive Laboratory Evolution
Source: Microb Biotechnol. 2025 Aug 10;18(8):e70208. doi: 10.1111/1751-7915.70208 (PMC12335938; doi:10.1111/1751-7915.70208)
Supplement: Supplementary file 2 — Figure S2: Biomass and by‐product concentrations of RE3, RE1, and LT1 autotrophic chemostat syngas cultures. [file MBT2-18-e70208-s009.pdf]

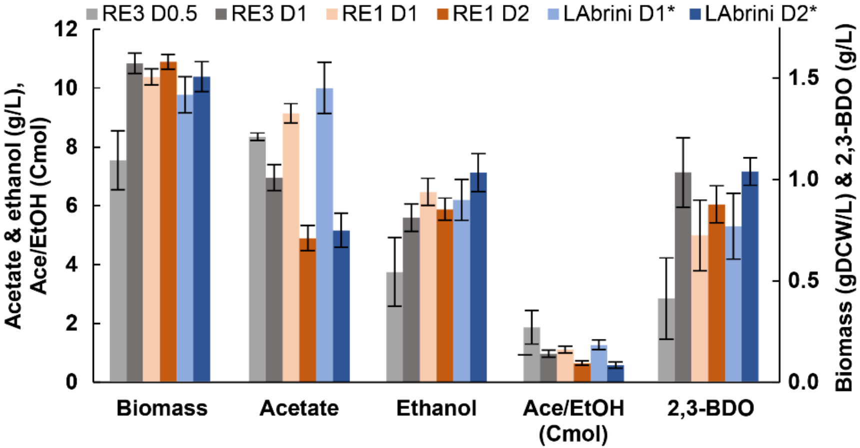

Figure S2. Biomass and by-product concentrations of RE3, RE1, and LT1 autotrophic chemostat syngas cultures. The number following D (dilution rate) denotes D value in day^{-1}. Bars show average ± standard deviation between bioreplicates (see methods for details). Asterisks behind names denote previously published data. 2,3-BDO, 2,3-butanediol; EtOH, ethanol; Ace/EtOH (Cmol), c-molar acetate to ethanol ratio; gDCW, gram of dry cell weight.
