# Supplementary material for: Reverse‐Engineered Gas‐Fermenting Acetogen Strains Recover Enhanced Phenotypes From Autotrophic Adaptive Laboratory Evolution
Source: Microb Biotechnol. 2025 Aug 10;18(8):e70208. doi: 10.1111/1751-7915.70208 (PMC12335938; doi:10.1111/1751-7915.70208)
Supplement: Supplementary file 3 — Figure S3: Correlation between acetate/ethanol (C‐mol) ratio and total specific electron consumption rate per biomass ((qH2 and qCO summed) × 2) across the whole bioreactor dataset. [file MBT2-18-e70208-s005.pdf]

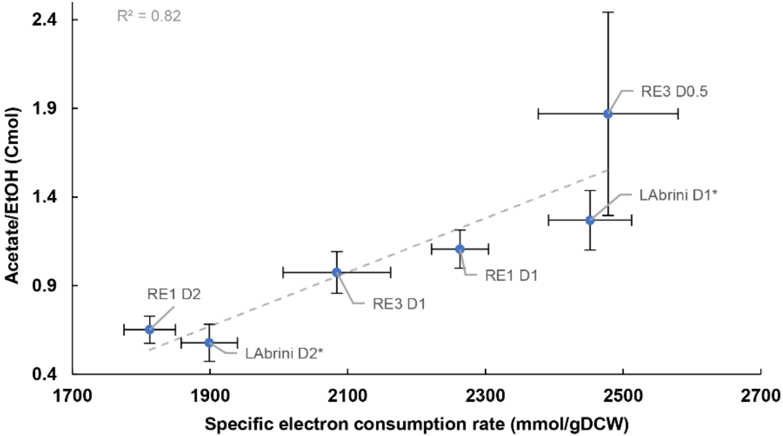

Figure S3. Correlation between acetate/ethanol (C-mol) ratio and total specific electron consumption rate per biomass ((q_{H2} and q_{CO} summed) × 2) across the whole bioreactor dataset. The number following D (dilution rate) denotes D value in day^{-1}. Bars show average ± standard deviation between bioreplicates (see methods for details). Asterisks behind names denote previously published data. EtOH, ethanol; Ace/EtOH (Cmol), c-molar acetate to ethanol ratio; gDCW, gram of dry cell weight.
