# Supplementary material for: Reverse‐Engineered Gas‐Fermenting Acetogen Strains Recover Enhanced Phenotypes From Autotrophic Adaptive Laboratory Evolution
Source: Microb Biotechnol. 2025 Aug 10;18(8):e70208. doi: 10.1111/1751-7915.70208 (PMC12335938; doi:10.1111/1751-7915.70208)
Supplement: Supplementary file 4 — Figure S4: In silico estimation of intracellular metabolic fluxes of central metabolism in syngas‐fermenting RE1, RE3, and LAbrini chemostats (see Figures 2 and 3 for experimental data). [file MBT2-18-e70208-s007.pdf]

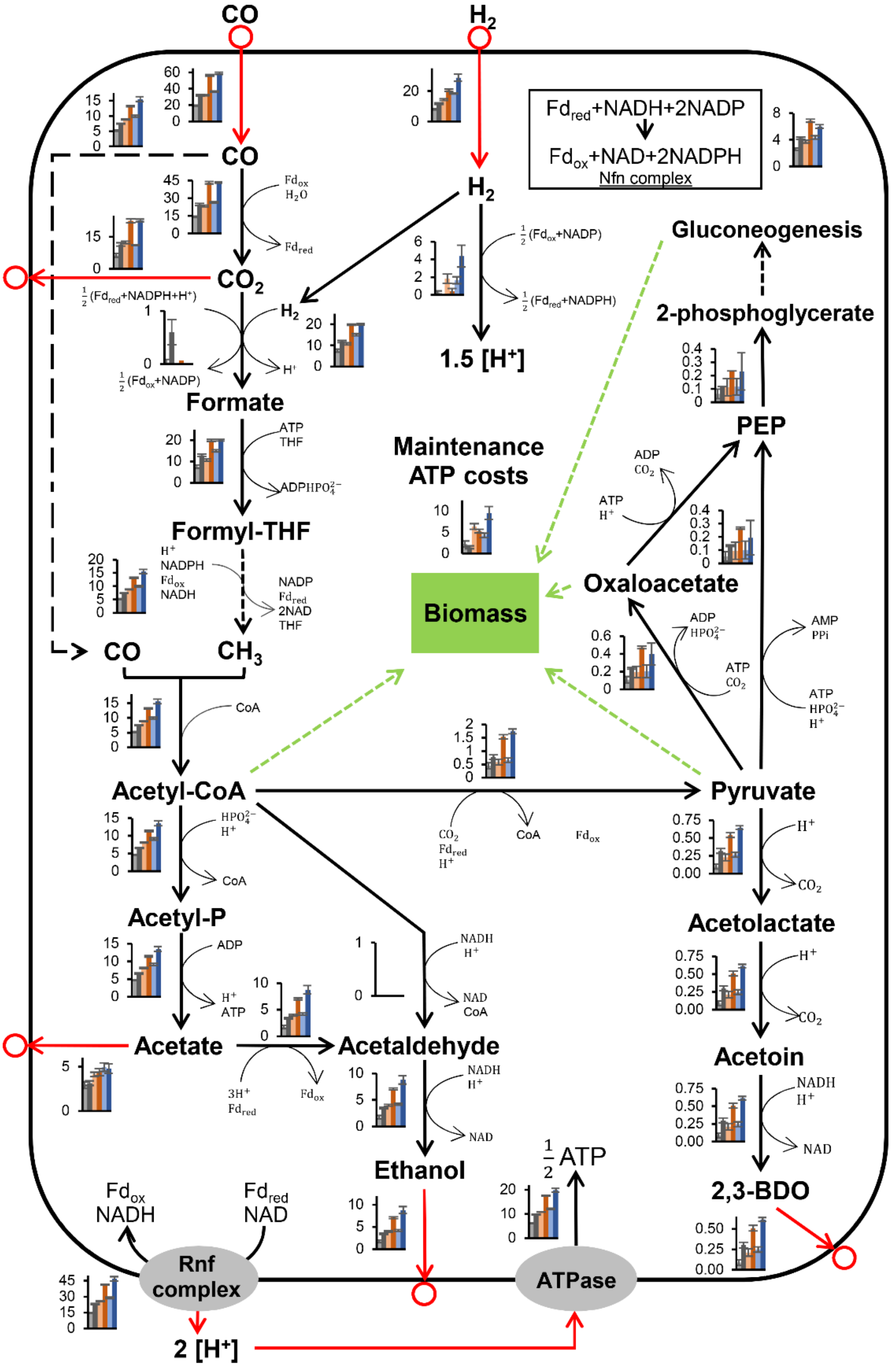

Figure S4. *In silico* estimation of intracellular metabolic fluxes of central metabolism in syngas-fermenting RE1, RE3, and LAbrini chemostats (see Figures 2 and 3 for experimental data). Bar chart colour coding is same as in Figure 2A and C: RE3 D0.5, gray; RE3 D1, dark gray; RE1 D1, light orange; RE1 D2, brown; LAbrini D1, light blue; LAbrini D2, dark blue. Fluxes (mmol/gDCW/h) are represented as average ± standard deviation between bioreplicates. Arrows show direction of calculated fluxes; red arrows denote uptake or secretion; dashed arrows denote a series of reactions. Cofactors used in the GEM iCLAU786 are shown. Flux into PEP from oxaloacetate and pyruvate is merged. The number following D (dilution rate) denotes D value in day^{-1}. OAA, oxaloacetate; PEP, phosphoenolpyruvate; THF, tetrahydrofolate; 2PG, 2-phosphoglycerate; 2,3-BDO, 2,3-butanediol. See Tables S3 & S4 for all modelling data and co-factor abbreviations.
