# Supplementary material for: Reverse‐Engineered Gas‐Fermenting Acetogen Strains Recover Enhanced Phenotypes From Autotrophic Adaptive Laboratory Evolution
Source: Microb Biotechnol. 2025 Aug 10;18(8):e70208. doi: 10.1111/1751-7915.70208 (PMC12335938; doi:10.1111/1751-7915.70208)
Supplement: Supplementary file 5 — Figure S5: Principal component analysis (PCA) of protein MS intensities for the reverse‐engineered strains RE1, RE2, RE3, and LAbrini and JA1–1. [file MBT2-18-e70208-s014.pdf]

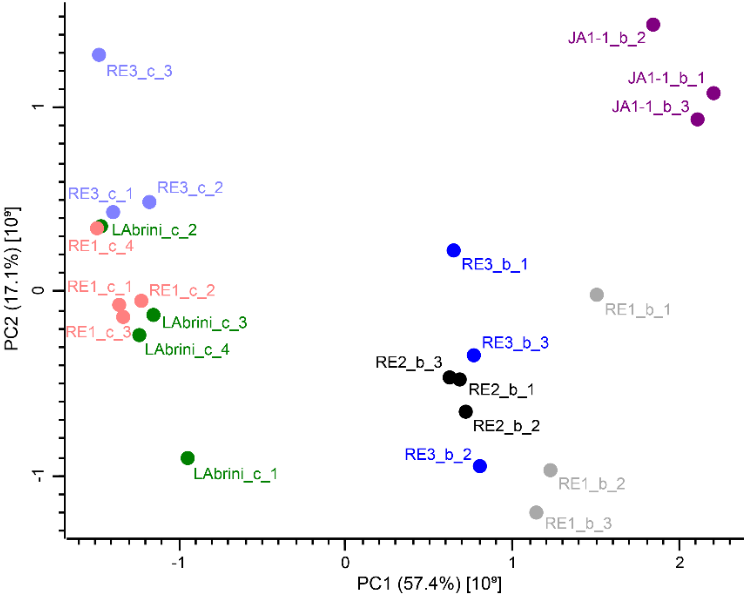

Figure S5. Principal component analysis (PCA) of protein MS intensities for the reverse-engineered strains RE1, RE2, RE3, and LAbrini and JA1–1. Data of RE1_b, RE2_b, and JA1-1 are from our previous work (Ingelman et al., 2023). Number at the end of data label denotes bioreplicate number. b, batch culture; c, chemostat; PC, principal component.
