# Supplementary material for: Reverse‐Engineered Gas‐Fermenting Acetogen Strains Recover Enhanced Phenotypes From Autotrophic Adaptive Laboratory Evolution
Source: Microb Biotechnol. 2025 Aug 10;18(8):e70208. doi: 10.1111/1751-7915.70208 (PMC12335938; doi:10.1111/1751-7915.70208)
Supplement: Supplementary file 6 — Figure S6: Multiple sequence alignment of proteins reverse‐engineered in this study with homologous proteins among Clostridia species. [file MBT2-18-e70208-s011.pdf]

A

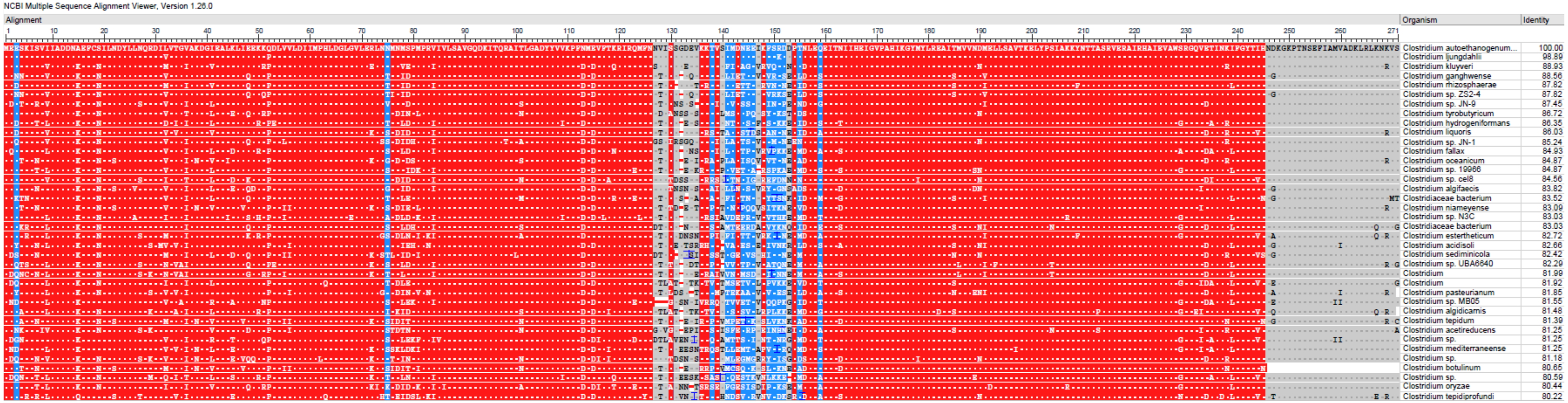
NCBI Multiple Sequence Alignment Viewer, Version 1.26.0
Alignment
Organism
Identity
Clostridium autoethanogenum... 100.00
Clostridium ljungdahlii 98.89
Clostridium kluyveri 88.93
Clostridium ganghwense 88.56
Clostridium rhizosphaerae 87.82
Clostridium sp. ZS2-4 87.82
Clostridium sp. JN-9 87.45
Clostridium tyrobutyricum 86.72
Clostridium hydrogeniformans 86.35
Clostridium liquoris 86.03
Clostridium sp. JN-1 85.24
Clostridium fallax 84.93
Clostridium oceanicum 84.87
Clostridium sp. 19966 84.87
Clostridium sp. cel8 84.56
Clostridium algifaecis 83.82
Clostridiaceae bacterium 83.52
Clostridium niameyense 83.09
Clostridium sp. N3C 83.03
Clostridiaceae bacterium 83.03
Clostridium estertheticum 82.72
Clostridium acidisoli 82.66
Clostridium sediminicola 82.42
Clostridium sp. UBA6640 82.29
Clostridium 81.99
Clostridium 81.92
Clostridium pasteurianum 81.85
Clostridium sp. MB05 81.55
Clostridium algidicarnis 81.48
Clostridium tepidum 81.39
Clostridium acetireducens 81.25
Clostridium sp. 81.25
Clostridium mediterraneense 81.25
Clostridium sp. 81.18
Clostridium botulinum 80.65
Clostridium sp. 80.59
Clostridium oryzae 80.44
Clostridium tepidiprofundi 80.22

B

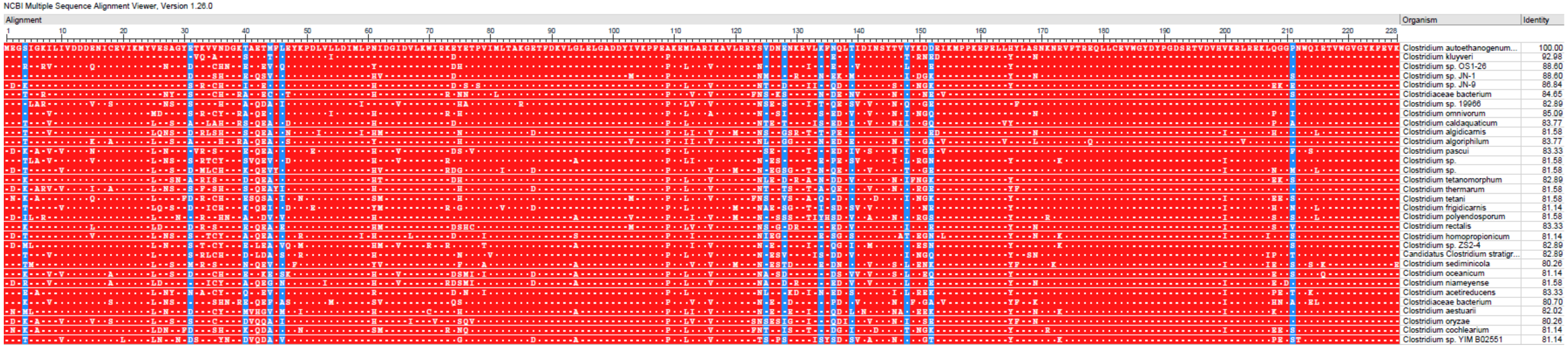
NCBI Multiple Sequence Alignment Viewer, Version 1.26.0
Alignment
Organism
Identity
Clostridium autoethanogenum... 100.00
Clostridium kluyveri 92.98
Clostridium sp. OS1-26 88.60
Clostridium sp. JN-1 88.60
Clostridium sp. JN-9 86.84
Clostridiaceae bacterium 84.65
Clostridium sp. 19966 82.89
Clostridium omnivorum 85.09
Clostridium caldaquaticum 83.77
Clostridium algidicarnis 81.58
Clostridium algoriphilum 83.77
Clostridium pascui 83.33
Clostridium sp. 81.58
Clostridium sp. 81.58
Clostridium tetanomorphum 82.89
Clostridium thermarum 81.58
Clostridium tetani 81.58
Clostridium frigidicarnis 81.14
Clostridium polyendosporum 81.58
Clostridium rectalis 83.33
Clostridium homopropionicum 81.14
Clostridium sp. ZS2-4 82.89
Candidatus Clostridium stratigr... 82.89
Clostridium sediminicola 80.26
Clostridium oceanicum 81.14
Clostridium niameyense 81.58
Clostridium acetireducens 83.33
Clostridiaceae bacterium 80.70
Clostridium aestuarii 82.02
Clostridium oryzae 80.26
Clostridium cochlearium 81.14
Clostridium sp. YIM B02551 81.14

C

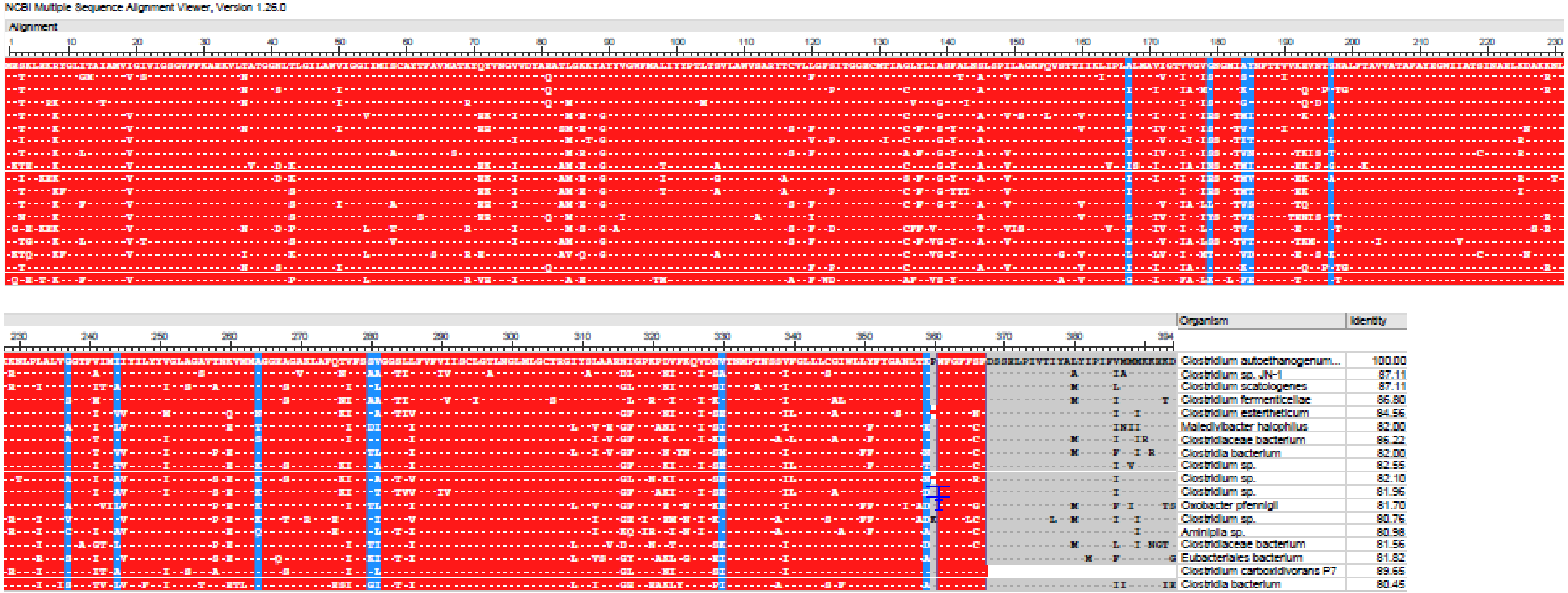

Figure S6. Multiple sequence alignment of proteins reverse-engineered in this study with homologous proteins among Clostridia species. (A) CLAU_3129, sporulation transcriptional activator Spo0A. (B) CLAU_1957, two component transcriptional regulator winged helix family. (C) CLAU_0471, amino acid permease. Obtained using NCBI blastp suite against ClusteredNR database. Search performed with default settings, results filtered with 80% identity cut-off and for class 'Clostridia'. Amino acid colouring is based on conservation: alignment columns with no gaps are coloured in blue or red where red indicates highly conserved positions and blue less conserved ones.
